# Gemfibrozil-Induced Intracellular Triglyceride Increase in SH-SY5Y, HEK and Calu-3 Cells

**DOI:** 10.3390/ijms24032972

**Published:** 2023-02-03

**Authors:** Cornel Manuel Bachmann, Daniel Janitschke, Anna Andrea Lauer, Tobias Erhardt, Tobias Hartmann, Marcus Otto Walter Grimm, Heike Sabine Grimm

**Affiliations:** 1 Experimental Neurology, Saarland University, 66421 Homburg, Germany; 2 Nutrition Therapy and Counseling, Campus Rheinland, SRH University of Applied Health Sciences, 51377 Leverkusen, Germany; 3 Physical Therapy, Campus Karlsruhe, SRH University of Applied Health Sciences, 76185 Karlsruhe, Germany; 4 Deutsches Institut für DemenzPrävention (DIDP), Saarland University, 66421 Homburg, Germany

**Keywords:** gemfibrozil, lipidomics, triglycerides, lipid droplets, phosphatidylcholine, carnitines, plasmalogens, lyso-phosphatidylcholine, Alzheimer’s disease, renal diseases, lung diseases

## Abstract

Gemfibrozil is a drug that has been used for over 40 years to lower triglycerides in blood. As a ligand for peroxisome proliferative-activated receptor-alpha (PPARα), which is expressed in many tissues, it induces the transcription of numerous genes for carbohydrate and lipid-metabolism. However, nothing is known about how intracellular lipid-homeostasis and, in particular, triglycerides are affected. As triglycerides are stored in lipid-droplets, which are known to be associated with many diseases, such as Alzheimer’s disease, cancer, fatty liver disease and type-2 diabetes, treatment with gemfibrozil could adversely affect these diseases. To address the question whether gemfibrozil also affects intracellular lipid-levels, SH-SY5Y, HEK and Calu-3 cells, representing three different metabolically active organs (brain, lung and kidney), were incubated with gemfibrozil and subsequently analyzed semi-quantitatively by mass-spectrometry. Importantly, all cells showed a strong increase in intracellular triglycerides (SH-SY5Y: 170.3%; HEK: 272.1%; Calu-3: 448.1%), suggesting that the decreased triglyceride-levels might be due to an enhanced cellular uptake. Besides the common intracellular triglyceride increase, a cell-line specific alteration in acylcarnitines are found, suggesting that especially in neuronal cell lines gemfibrozil increases the transport of fatty acids to mitochondria and therefore increases the turnover of fatty acids for the benefit of additional energy supply, which could be important in diseases, such as Alzheimer’s disease.

## 1. Introduction

**Figure 1:**
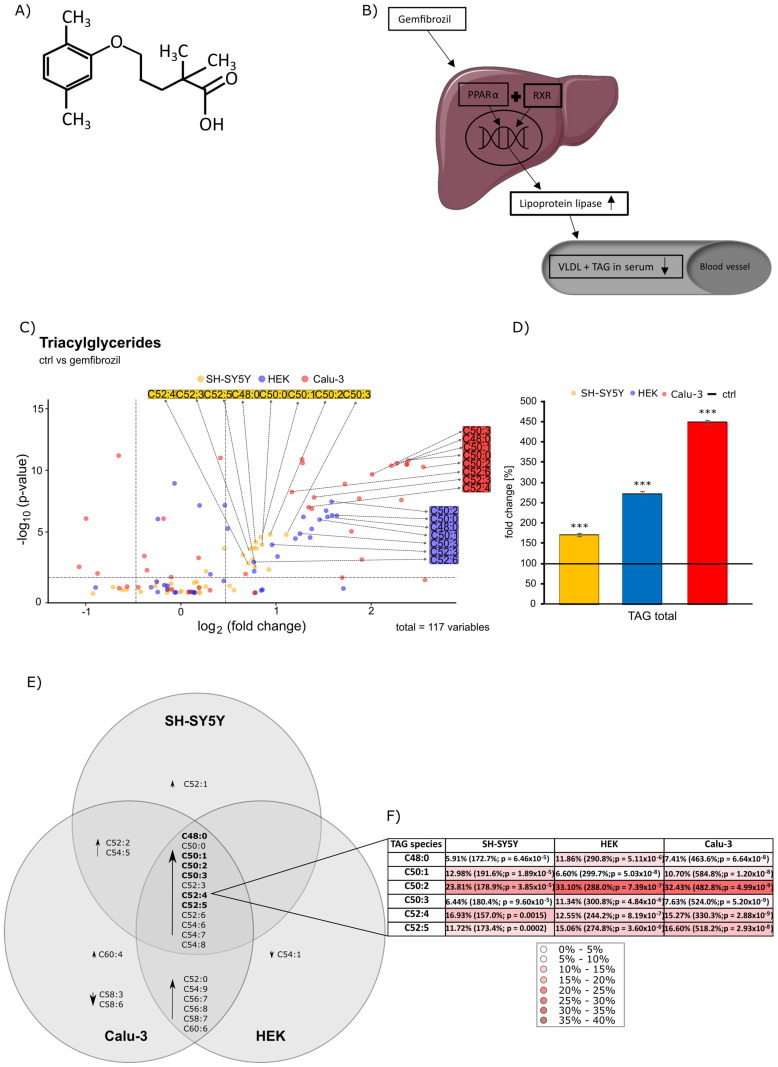
(**A**) chemical structure of gemfibrozil (**B**) Schematic representation of the mechanism of action of gemfibrozil in interaction with PPARα and RXR and their gene regulatory influence on lipoprotein lipase and the resulting reduction in TAG and VLDL levels in blood plasma. (**C**) changed triacylglyceride (TAG) levels in SH-SY5Y, HEK and Calu-3 cells after gemfibrozil treatment compared to cells treated with the solvent control (ethanol) are illustrated in the volcano plot, where the fold change (x-axis) of each of the 39 examined TAG species (dots) was plotted against the corresponding logarithmic *p*-value (y-axis). All three cell lines are shown in one volcano plot with three different colors: SH-SY5Y in orange, HEK in blue and Calu-3 in red. (**D**) The bar chart shows the relative fold change, by calculating the significance level using the students *t*-test of gemfibrozil-treated cells against the control per cell line, of all measured TAG species in the three cell lines after gemfibrozil treatment in comparison to treatment with the solvent control (marked as horizontal line and set as 100%), with *** *p* ≤ 0.001. (**E**) Venn diagram of TAG species is illustrated. All species which showed a significant alteration are displayed belonging to their respective cell line. Major species of these TAGs are written in bold letters. (**F**) The heatmap shows the respective major species of all TAG species examined that are most represented in percentage within a cell line. According to their percentage frequency, they were highlighted with different intensities of red coloration, whereby the higher the percentage, the more intense the red coloration. The respective effect size is indicated in parentheses and the shown *p*-values were calculated using the students *t*-test of gemfibrozil-treated cells against the control per cell line.

The drug gemfibrozil has been used since 1982 to reduce elevated triglyceride and cholesterol levels in the blood plasma [1]. Along with bezafibrate and fenofibrate, it belongs to the fibric acid derivatives (FAD’s) [2]. It is indicated as a supportive treatment in addition to diet or ineffective drug therapy with other hypolipidemic effects in severe hypertriglyceridaemia with and without lowered high-density lipoprotein (HDL) cholesterol level, in mixed hyperlipidaemia when a statin is contraindicated or not tolerated or in primary hypercholesterolaemia in statin intolerance [3]. All FAD’s cause a reduction in triacylglycerides ((TAG) 40–60%), very low-density lipoproteins (VLDL), low-density lipoproteins (LDL (10–20%)) and elevates the level of HDL (5–20%) [4,5]. Chemically, gemfibrozil is oxidized by a ring methyl group and forms a hydroxymethyl and a carboxymetabolite (see Figure 1A). A study on cortical neurons in mice showed that gemfibrozil passes the blood–brain barrier and thus also enters the central nervous system [6].

The main effect of all fibrates is due to their agonism at peroxisome proliferative-activated receptor-alpha (PPARα). Peroxisome proliferative-activated receptors (PPARs) in general belong to the family of nuclear receptors that influence gene regulation of carbohydrate and lipid metabolism [3]. PPARs are expressed in many tissues, such as adipocytes, muscles and endothelial cells [4]. PPARα is usually highly expressed in metabolically active tissues, such as liver, heart, skeletal muscle, intestinal mucosa and brown adipose tissue. In addition to these tissues, expression of PPARα takes places in kidney, lung and brain [4,7,8,9].

Binding of gemfibrozil to PPARα results in a conformational change, which is followed by heterodimer formation with another nuclear receptor, retinoid X receptor (RXR), involving recruited coactivators. This complex binds to PPAR response elements, which then dock to the promoter region of the target genes, thus regulating gene expression [3]. VLDL levels are lowered because gemfibrozil promotes the degradation of VLDL triglycerides in hepatocytes by increasing the activity of extracellular lipoprotein lipase, which subsequently leads to a decrease in serum triglycerides. Accordingly, activation of PPARα promotes lipolysis of VLDL and triglycerides in blood (schematic representation in Figure 1B exemplary for TAG and VLDL). Additionally, gemfibrozil decreases VLDL prediction while inhibiting the synthesis and increasing the clearance of VLDL carrier apolipoprotein B [4]. However, the fundamental mechanism of action is not fully elucidated until now [5].

Additionally, it was reported, that gemfibrozil can increase the level of plasma HDL cholesterol concentration and may, therefore, be potent to minimize the cardiovascular disease outcome [10].

Gemfibrozil is recommended at a dosage of 600 mg twice daily 30 min before meals in the morning and evening and is quickly and completely absorbed after ingestion. After a single dose of gemfibrozil 600 mg, a peak plasma concentration of 20 mg/L (=79.89 µmol/L) is reached after 1 to 2 h. A steady state is reached within 1 to 2 weeks when gemfibrozil 600 mg is taken twice daily. It is 97% bound to serum albumin and is mostly excreted in the urine (70%) by binding to glucuronide conjugates after metabolized in the liver [5]. The mean elimination half-life of gemfibrozil is 7.6 h. It may take several months to achieve the maximum response from gemfibrozil. It is indicated in all patients with dyslipidemia of type II or higher, and in patients with significantly elevated triglyceride levels (>7.5g/L) and increased risk of pancreatitis [5].

The most frequently described side effects concern the gastrointestinal tract such as diarrhea and nausea. Skin rash, musculoskeletal complaints (myalgia, rhabdomyolysis) and cholelithiasis have also been described. Apart from serious renal or hepatic insufficiency, pregnancy and breastfeeding are the most frequent contraindications to the use of gemfibrozil, along with diseases of the biliary system [11]. In addition, it is known that parallel intake of other pharmaceuticals (e.g., sulfonylureas and vitamin K antagonists) can lead to an increased effect of gemfibrozil. In patients with additional therapy of vitamin K antagonists, the dose of the coumarin derivatives must be adjusted and possibly the prothrombin time checked regularly. An increased incidence of rhabdomyolysis has been observed with combined intake of statins and gemfibrozil [11].

Due to the expression of PPARα in many different tissues it can be assumed that gemfibrozil affects numerous areas of the organism. For several years now, knowledge and studies on lipid droplets (LDs) have been increasing. LDs are storage organelles that have a significant influence on lipid and energy homeostasis. They consist of a hydrophobic core with neutral lipids, mainly triglycerides and cholesterol, and are surrounded by a lipid monolayer, where a large number of proteins are stored. They are in close contact with other cell organelles and have a very dynamic effect. In the meantime, both physiological and pathophysiological participations of lipid droplets are known [12].

Numerous studies point to an additional effect of gemfibrozil on other diseases. For example, gemfibrozil has been shown to have a positive effect on preventing the development of neurodegenerative diseases, e.g., Alzheimer’s Disease [13]. In the Helsinki Heart Study, it was shown that long-term therapy with gemfibrozil significantly reduces the occurrence of cardiac events [14].

Over the years, both the efficacy of gemfibrozil as a fibrate for lowering blood lipids and its mechanism of action have been studied in great detail. In contrast, there is little data on how gemfibrozil affects different organs and their lipid composition in peripheral cells. To address the question of how gemfibrozil affects other organs and influences their lipid composition, we focused in this study on three different cell lines in which LDs accumulation is of great pathophysiological interest. In neuronal tissue, increased LDs accumulation is associated with the development of neurodegenerative diseases, such as Alzheimer’s or Parkinson’s disease. In the development of diabetic nephropathy, lipid accumulation is also implicated as a cause of renal dysfunction. Especially in the context of the current COVID-19 pandemic, there has been additional interest in lipid changes in lung diseases. For this reason, in this study we focused on SH-SY5Y cells, representing a neuronal cell line, HEK cells, representing a renal cell line and Calu-3 cells, representing a pulmonary cell line. Cells were incubated with gemfibrozil, then analyzed semi-quantitatively by mass spectrometry.

## 2. Results

To find out whether gemfibrozil influences the lipid composition of different cell-types in the body, human neuroblastoma cells (SH-SY5Y), human embryonic kidney cells (HEK) and human lung cells (Calu-3) were incubated with 100 µmol/L gemfibrozil for 72 h (24 h + 24 h +24 h). This concentration, which is in the supraphysiological range, represents an often-used dosage in studies with cell incubation [15,16] and showed no cytotoxicity (see Supplementary Figure S1A). Subsequently, over 178 lipids were analyzed by mass spectrometry. These analyzed lipids could be divided in different classes. Phospholipids including Phosphatidylcholines (PCaa), Phosphatidylcholine plasmalogens (PCae), lyso-Phosphatidylcholines (lyso-PC) and sphingomyelin (SM) on the one hand and TAGs as neutral lipids and lipids involved in the mitochondrial carnitine shuttle system (carnitine (C)) on the other hand. Carnitines play an essential role especially in fat metabolism, as they are responsible for the transport of fatty acids (FA) between the cytosol and the mitochondria, where β-oxidation takes place [17].

**Figure 2:**
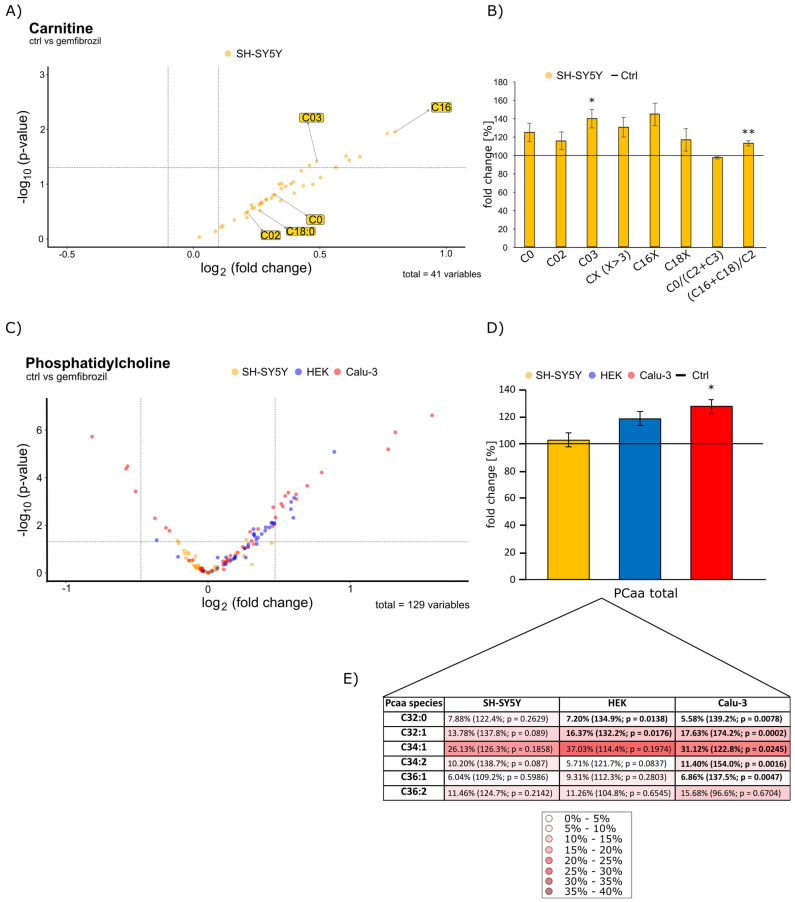
(**A**) Changed carnitine levels in SH-SY5Y cells after Gemfibrozil treatment compared to cells treated with the solvent control (ethanol) are illustrated in the volcano plot, where the fold change (x-axis) of each of the 41 examined carnitine species (dots) was plotted against the corresponding logarithmic *p*-value (y-axis). The volcano plot is structured as described in detail in the legend of Figure 1**.** (**B**) The bar chart shows the relative fold changes in C0, C2, C3, CX (X > 3) and (C16 + C18)/C2 ratio in Gemfibrozil treated SH-SY5Y cells compared to cells treated with the solvent control (ethanol), which is represented as the horizontal line and set as 100%. The obtained data were calculated by using the students *t*-test of gemfibrozil-treated cells against the control of SH-SY5Y-cells, with * *p* ≤ 0.05 and ** *p* ≤ 0.01. (**C**) Changed Phosphatidylcholine (PCaa) levels in SH-SY5Y, HEK and Calu-3 cells after gemfibrozil treatment compared to cells treated with the solvent control (ethanol) are shown in the volcano plot, where the 43 analyzed PC aa species are plotted for each cell line in the same way as in the other volcano plots on the top. (**D**) A bar chart shows the relative fold change of PCaa total for each cell line treated with gemfibrozil and compared to cells treated with the solvent control (ethanol) marked as horizontal line (and set as 100%). The obtained data were calculated by using the students *t*-test of gemfibrozil-treated cells against the control of each cell line, with * *p* ≤ 0.05. (**E**) The heatmap shows the respective major species of all PCaa species examined that are most represented in percentage within a cell line. According to their percentage frequency, they were highlighted with different intensities of red coloration, whereby the higher the percentage, the more intense the red coloration. The respective effect size is indicated in parentheses and the shown *p*-values were calculated using the students *t*-test of gemfibrozil-treated cells against the control per cell line.

Deuterated standards for each lipid class were used to normalize the data obtained. Then, these data were plotted as x-fold change compared to the control group. The observed relative lipid changes in gemfibrozil-treated cells of all three cell types compared to the solvent-treated control group are shown in Figure 1 and Figure 2 for triglycerides, carnitine and PCaa in the form of volcano plots, respectively. Here, the x-fold change (abscissa) is plotted against the *p*-value (ordinate). The horizontal line in the diagram stands for a *p*-value of 0.05, which was set as statistical significance, and the two vertical lines show the average standard error of the mean (SEM), per lipid class from each cell line. The adjacent bar graphs show the total percentage changes of a lipid species induced by gemfibrozil on the different cell lines compared to their respective control, unless otherwise stated.

### 2.1. Mass Spectrometry Analysis of Triglycerides in Gemfibrozil-Treated SH-SY5Y-, HEK- and Calu-3 Cells

TAGs belong to the neutral fats and perform a role in energy and fat metabolism [18]. In everyday medical practice, TAGs are measured in the blood, as elevated values indicate a lipometabolic disorder and/or obesity. They are also a cardiovascular risk factor [19]. Chemically, TAGs are triple esters of the trivalent alcohol glycerol with three acid molecules [20]. They are an important marker for energy metabolism and can act as energy storages by forming lipid droplets in the cytosol of a cell [20]. The fatty acids derived from the TAGs can be used in the mitochondria for energy production. For this purpose, each individual fatty acid is split off and bound to carnitine. In our study, SH-SY5Y, HEK and Calu-3 cells incubated with gemfibrozil were compared with their respective control groups, then relative changes of each cell line were compared with each other. The observed mass spectrometric changes of a single lipid can on the one hand be due to the head group of a lipid, but they can on the other hand be caused by the fatty acid bound to the backbone of the lipid.

Interestingly, gemfibrozil treatment resulted in significantly increased intracellular TAG levels in all three cell lines (SH-SY5Y *p* ≤ 0.001; HEK *p* ≤ 0.001; Calu-3 *p* ≤ 0.001, see Figure 1D). There is a clear rightward shift within the volcano plot (Figure 1C).

In SH-SY5Y cells, gemfibrozil treatment resulted in an increase in 25 out of the 39 TAG species examined and a significant increase to a total of 170.3 ± 4.9% (*p* ≤ 0.001) (Figure 1D). Of these 25 elevated TAG species, 15 TAG species were significantly increased by gemfibrozil. In contrast, 14 TAG species were reduced, with no species being significantly decreased (see Supplementary Materials Figure S1B).

Similar results can be found when HEK cells were treated with gemfibrozil compared to the control group (Figure 1C,D). 23 out of the 39 analyzed TAG species were elevated and an overall significant increase in all TAG species in total compared to the control group to 272.1 ± 6.0% (*p* ≤ 0.001) (Figure 1D) was detected. Of these 23 increased TAG species, 18 TAG species were significantly increased by gemfibrozil (see Supplementary Figure S1B). Eleven TAG species were decreased in HEK cells, but no one reached the significance level.

The strongest rightward shift in TAG species with gemfibrozil treatment compared to the control group was documented for Calu-3 cells (Figure 1C). There was an overall highly significant increase in all TAG species in total compared to the control group to 448.1 ± 3.9% (*p* ≤ 0. 001) (Figure 1D). Within the 39 TAG species examined, 27 were elevated and 12 were decreased. Of the 27 elevated TAG species, a total of 23 were significantly elevated. Of the 12 decreased species, 4 species reached the significance level (see Supplementary Materials Figure S1B).

The Venn diagram shown in Figure 1E illustrates all significantly altered TAG species that occurred in the three cell lines examined. All species with a significance of *p* ≤ 0.05 were considered. Interestingly, there are 12 TAG species that were significantly increased in all three cell lines. These are TAG C48:0, TAG C50:0, TAG C50:1, TAG C50:2, TAG C 50:3, TAG C52:3, TAG C52:4, TAG C52:5, TAG C52:6, TAG C54:6, TAG C54:7 and TAG C54:8. In addition to these species, there are a number of other TAGs that were significantly altered by gemfibrozil treatment in the cell lines. TAG C52:0, TAG C54:9, TAG C56:7, TAG C56:8, Tag C58:7 and TAG C60:6 were significantly increased and altered in HEK and Calu-3 cells. TAG C52:2 and TAG C54:5 were similarly altered in SH-SY5Y and Calu-3. TAG C52:1 was significantly increased only in SH-SY5Y cells and TAG C54:1 only in HEK cells. In general, most TAG species were significantly altered in lung cells. In addition to those mentioned above, TAG C60:4 was significantly increased and, interestingly, two species were significantly decreased (TAG C58:3 and TAG C58:6) in Calu-3 cells. The heatmap 1F shows a selection of representatives of the major species of triglycerides after gemfibrozil treatment that are most highly represented in percentage terms. Based on the species in the Venn diagram that were significantly increased, the major representatives were listed with their species’ percentage of the total percentage of all TAG species. The respective effect sizes are written in parentheses. Over all three cell lines examined the major species present is C50:2, which is present in HEK cells with a percentage of 33.10%.

### 2.2. Gemfibrozil-Induced Changes of Carnitine Species in SH-SY5Y, HEK and Calu-3 Cells

Carnitines play a major role in fatty acid transport from the cytosol across the inner mitochondrial membrane into the mitochondria, where fatty acid degradation is regulated by β-oxidation [17]. Since we found a significant increase in intracellular TAGs by gemfibrozil treatment in our study, carnitines have been focused consequently. In the volcano plot in Figure 2A, the distributions of the 41 carnitine species examined are plotted for SH-SY5Y cells, only. In SH-SY5Y cells incubated with gemfibrozil, all carnitines examined are exclusively elevated, with individual species reaching the significance level (C0, *p* = 0.0385 and (C16 + C18)/C2, *p* = 0.004). In comparison, the distribution of carnitine species in kidney and lung cells is not clearly divided. In kidney cells and in lung cells, there are increased and significantly decreased species (see Supplementary Materials Figure S1C,D). However, since β-oxidation cyclically degrades carbon atoms and yields energy in the process [21], C0 as main carrier for the FAs, the end products, C02 and C03, and the two major representatives, C16 and C18, are of particular interest for evaluating the metabolism, with the ratio of (C16 + C18)/C2 especially providing a meaningful measure of β-oxidation. CX (X > 3) is representative for all acyl-carnitines with more than three carbon atoms, that is increased in SH-SY5Y after treatment with gemfibrozil to 130.7% ± 10.7% (Figure 2B). Therefore, we have plotted in Figure 2B in the form of a bar chart the respective distribution of the main species for SH-SY5Y cells compared to the respective control. The control was set to 100 per cent and shown as a horizontal line. These results suggest that increased FAs should be degraded in neurons in response to the significantly increased accumulation of intracellular TAGs by β-oxidation.

Interestingly, opposite results are seen in both, gemfibrozil-treated kidney and lung cells. C03, the end product of odd fatty acids, is even significantly decreased in HEK cells to 57.7% (*p* ≤ 0.001). In the lung cells, it does not reach the significance level, but is nevertheless reduced to 73.6% (*p* = 0.1549) (see Supplementary Figure S1D). The results suggest that the fatty acids are not yet processed by β-oxidation in response to the massive TAG increase.

### 2.3. Gemfibrozil-Induced Changes of Phosphatidylcholine (PCaa) Species in SH-SY5Y, HEK and Calu-3 Cells

The most important components of biological membranes are phosphoglycerides. Phosphatidylcholines (PCaa), which are considered one of the main representatives (30–35%) [22] of phosphoglycerides, are ubiquitously present in all cell membranes and also perform an important role in signal transduction [23]. Interestingly, PCaa make up approximately 9–10% of the dry mass of the human brain [22]. In addition, as monolayers, they represent the hydrophilic component of the outer shell of lipid droplets [24]. Their chemical structure consists of a glycerol linked to two FAs on the one hand and to a choline via a phosphate group on the other. In our study, we show the distribution of the 43 PC aa species examined again in the form of a volcano plot (see Figure 2C). Here, a slight rightward shift of the PC aa species of all three cell lines can be documented. Since we assume that the massive increase in TAGs is stored intracellularly in the form of so-called lipid droplets, the PCaa species are of additional interest as monolayers of them [24]. To illustrate this, we have plotted a bar chart (see Figure 2D) with the total of all PCaa to the right of the volcano plot. Here, analogous to the massive TAG increase, there is also an increase in PCaa species in all cell lines with similar effect strength (Calu-3 > HEK > SH-SY5Y). In the SH-SY5Y cells, there is an increase to 103.0% ± 5.0%. In the HEK cells, the PCaa total increases to 118.8% ± 4.9% and for the Calu-3 cells to 127.6% ± 6.8% (*p* = 0.0127). Heatmap 2E shows a selection of representatives of the major species of phosphatidylcholines that are most highly represented in percentage terms. Based on all PCaa species examined in this study, the major representatives were listed with their species’ percentage of the total percentage of all PCaa species. The respective effect sizes are written in parentheses. Over all three cell lines examined, the major species are C32:1 and C34:1.

### 2.4. Alterations on Other Phospholipids (Phosphatidylcholine Plasmalogens, Lyso-Phosphatidylcholine and Sphingomyeline) by Gemfibrozil Treatment

In this section, we would like to give only a brief overview of the other examined phospholipids altered by gemfibrozil. For this purpose, Table 1 shows the respective percentage changes to their respective controls on each cell line of all lipid classes measured by mass spectrometry and a detailed group analysis for the examined lipid classes can be found in the Supplementary Tables S6–S11.

Phosphatidylcholine plasmalogens (PCae) also belong to the membrane glycerophospholipids [25] and consist of a fatty alcohol containing a vinyl ether bond at the sn-1 position and enriched with polyunsaturated fatty acids on the sn2 position [25]. They account for up to 20% of the total phospholipid mass in humans [25]. In our study, all 39 PCae species examined were increased in SH-SY5Y cells to a total of 129.9% without statistical significance. In HEK (98.1%) and in Calu-3 cells (84.4%), there is an overall decrease in PCae species.

Lyso-PC are formed by degradation from phosphatidylcholines by the activity of phospholipase A2 by cleaving one of the fatty acid groups [26]. They occur in small amounts in the plasma membrane (<3%) and in free form in the blood plasma, where they are a harbinger of atherosclerosis through contact with the endothelium [22,26]. Our study shows an increase across all three cell lines in the 22 lyso-PC species examined (SH-SY5Y Lyso-PC total: 121.0%, HEK Lyso-PC total: 126.8%, *p* = 0.0062 and Calu-3 Lyso-PC total: 143.2%, *p* = 0.0172%), being significant for HEK and Calu-3 cells

Sphingomyelins are also present in biological membranes [27]. In the brain in special, their proportion is of great importance, as they make up the main component of the myelin sheaths. SM perform an important role in neuronal signal transduction. In particular, their interaction with ceramides is of special interest for the development of AD, since deregulation of the SM/ceramide signaling cascade leads to synaptic dysfunction, neuroinflammation and neuronal apoptosis [28,29,30]. In the total of 15 species studied here, there was an increase in all three cell lines. The greatest increase in SM is seen in SH-SY5Y cells. Here, there is an overall increase to 126.6% without statistical significance. However, there was also an increase in HEK cells (121.0% with *p* = 0.047) and lung cells (110.8%).

## 3. Discussion

Gemfibrozil is a drug that has been established for 40 years to lower elevated triglyceride, VLDL and LDL levels [1]. In addition, it has been shown in studies to increase HDL concentrations and thus has a legitimate place in lipid-lowering therapy [14]. The mechanism of action for lowering elevated triglyceride levels in blood plasma is currently well understood, even though it is not fully explained yet. However, it is not yet clear what happens to the degraded triglycerides. After degradation, are they metabolized and excreted via the renal or hepatobiliary system or is there an intracellular uptake of triglycerides into other organs?

To address this issue, in this study we focused on the question of how individual cells of different organs cope with the supply of gemfibrozil and how the lipid profile of the cells changes. Considering that the indication of gemfibrozil often involves multimorbid patients with a broad spectrum of previous diseases, the question also arises how this influence of gemfibrozil would affect organs concerned. Representing three organs, we focused on a neuronal cell line (SH-SY5Y), a renal cell line (HEK) and a pulmonary cell line (Calu-3) in this study.

The lipidomics approach explained here was used to identify similarities and differences between the different cell lines, which in addition may also partly allow conclusions to be drawn about the different tissues. In general, we found that when considering neutral lipids with respect to TAGs, there was a significant increase across all cell lines, whereas when subsequently considering carnitines, there were clear differences between neurons on the one hand and kidney and lung cells on the other. When phospholipids are considered, a similar effect is generally seen across all three cell lines examined. TAGs were most markedly increased in gemfibrozil-treated lung cells. Subsequently a significant TAG increase is also seen in the renal and neuronal cells. Consistent with these data obtained in this study, Baldo et al. also demonstrated a gemfibrozil-induced intracellular TAG increase in human skin fibroblasts [31].

Investigating the carnitine species, clear differences could be found. In the kidney and lung cells, there are reduced carnitine levels with even a significantly decreased formation of C03, as the end product of the odd-numbered fatty acids of β-oxidation, for the kidney cells. Thus, it suggests that the excess of fatty acids by triglycerides is not affected by β-oxidation after 72 h. In contrast, a general increase in carnitines is seen in neurons, highlighted in particular by the significant increase in C03 and the ratio of (C16 + C18)/C2. These results suggest that the boosted β-oxidation in the mitochondria of neurons is attempting to compensate for the oversupply of TAGs. These findings are consistent with the fact that TAGs are generally metabolized more frequently in the brain for energy [32].

Phospholipids are essential to build membranes. Thus, they are also required for the membranes of lipid droplets. The main components of the monolayers of lipid droplets are phosphatidylcholines [24]. Our results show a general increase in PCaa levels in all three cell lines studied, with the magnitude of appearance being analogous to the TAG increase within each cell line (Calu-3 > HEK > SH-SY5Y). This increase can be explained by the fact that the PCaa in the form of monolayers pack the TAGs and, thus, there is a marked increase in intracellular LDs in nerve, kidney and lung cells.

The indications for the use of gemfibrozil in clinical practice are nowadays defined in detail [10]. Frequently, patients are considered who, due to their elevated blood triglyceride and VLDL levels, also have other comorbidities and thus additional pre-diseases of other organs exist. From this point of view, the aspects of the massively significant increase in intracellular TAG levels, which probably lead to an increase in LDs in nerve, kidney, and lung cells, obtained in this study, have to be put into a special context. Pre-existing conditions in these organ systems, including increasing adiposity, could be further triggered by long-term gemfibrozil use.

Lipid droplets have gained importance in recent years, both in physiological and pathophysiological terms, because they have a status similar to that of cell organelles [12,33]. Their biogenesis takes place at the endoplasmic reticulum, where smaller lipid aggregates are formed and are then sequestered in sac form and released to the cytosol. They are composed primarily of TAG and cholesterol esters in the core, surrounded by a simple phospholipid layer [12,33]. A variety of proteins are incorporated into this membrane, including perilipins, which regulate the stimulation of hormone-sensitive lipase as an important component of lipolysis and is essential for the biogenesis of LDs [33] or the membrane-associated protein CGI-58 (co-activator comparative gene identification 58), which is a potent activator of adipose triglyceride lipase (ATGL) [34]. Thus, via these proteins, LDs influence the energy and fat metabolism of the organism. LDs also play an important role from a pathophysiological point of view. An accumulation of intracellular LDs is described in a variety of different diseases. Thus, they are a component in the development of inflammation, through the influence on the control of the synthesis and secretion of inflammatory mediators [35]. Enzymes involved in eicosanoid formation in cells are localized in LDs. In addition, and through their influence on the energy metabolism of a cell, LDs are directly related to cancer [35]. Diseases caused by chronic inflammation or increased intracellular energy demand thus seem to be directly related to lipid droplets, too.

As early as 1907, Alois Alzheimer described microscopically conspicuous “fat droplets” in his study of patients with Alzheimer’s disease (AD) [36]. These have since been defined as lipid droplets and, in addition to the formation of intracellular tau fibrils and the extracellular deposition of β-amyloid plaques, appear to have a significant effect on the development of AD [37,38]. Meanwhile, numerous studies indicate that the accumulation of lipid droplets in the brain stimulate the process of chronic neuronal inflammation and are thus causes of neurodegenerative diseases, such as AD or Parkinson’s disease, among others [37,39,40]. Aggregated LDs in glial cells accelerate neurodegeneration in Drosophila [41]. Increased accumulation of LDs has also been confirmed in the Parkinson’s disease mouse model [42]. In the increasingly aging brain, there is a rising accumulation of LDs in microglia. This accumulation results in defective phagocytosis and increased production of reactive oxygen species and increased secretion of proinflammatory cytokines [43]. In addition, extensive lipid changes have been found in the brains of Alzheimer’s patients and in Alzheimer’s animal models. These include changes in total phospholipid content including changes in phosphatidylcholine, plasmalogens, sphingomyelin, cholesterol, and others [27,44,45]. Furthermore, lipids are discussed to affect the release of amyloid-β-peptide (Aβ) from the amyloid precursor protein and Aβ degradation [46,47]. The data obtained in the present study also show a significant increase in TAGs in the SH-SY5Y cells, which could result in increased LD accumulation. However, since the carnitines responsible for mitochondrial shuttling of FAs are also increased and, with C03 and the ratio of (C16 + C18/C2), even two main features of β-oxidation are significantly increased, it can be assumed that β-oxidation also proceeds at an increased rate in the neurons under gemfibrozil treatment. Due to the increased β-oxidation, more fatty acids are available for energy production. Since there is an energy deficit in the brain, especially in Alzheimer’s disease, gemfibrozil can probably be attributed a protective effect in neurodegenerative diseases, what has already been demonstrated in other studies [14,48,49].

LDs also appear to perform a role in the development of renal diseases. For example, diabetic nephropathy is associated with an increasing intrarenal accumulation of lipid droplets [50]. This intrarenal TAG increase, especially in glomerulus cells and cells of the proximal tubule, seems to be the cause of renal function loss [51,52,53]. This progressive lipid accumulation under inadequately controlled diabetic metabolic conditions leads to increasing glomerulosclerosis, which further promotes renal dysfunction [54]. Although it is not yet clear whether increased intrarenal lipid accumulation is a cause or consequence of diabetic nephropathy, it can at least be stated that it is associated with increased renal function loss.

Increased levels of lipid droplets in the lung are associated with many pulmonary diseases. Thus, accumulation of LDs in the lung also performs a role in current SARS-CoV-2 infection. In 2020, it was shown that viruses in general, and thus also the SARS-CoV-2 virus, rely on LDs as an assembly form [55]. Through this assembly form, LDs influence viral replication and pathogenesis of SARS-CoV-2. However, LDs appear to play a role not only in acute lung inflammation, but also in chronic inflammation. In addition to the viral assembly platform, the fatty acids of TAGs are the only source of energy for Mycobacterium tuberculosis [56]. Furthermore, tuberculosis patients who have a high content of LDs are associated with a higher rate of poor treatment outcomes [57]. In patients suffering from cystic fibrosis, dysregulation and accumulation of LDs has been associated with aggravation of the disease [58]. In addition to these inflammatory diseases of the lung, LDs also appear to have an impact on the development and aggravation of lung cancer. That lipid metabolism is directly related to cancer metabolism and is now well-known and sufficiently discussed. Elevated levels of LDs are associated with tumor development and progression of lung cancer, with accumulation of LDs enhancing cancer aggressiveness and promoting increasing resistance to chemotherapeutic agents. In gland-rich tissues, such as the lungs, Prostaglandin E2 (PGE-2) performs an important role in promoting tumor growth. LDs are known to be the site of synthesis of PGE-2. Here, Cyclooxygenase-2 is recruited and used to synthesize PGE-2 [35,59].

PPARα is a ligand-activated transcription factor responsible for the expression of a variety of different genes in lipid metabolism. In the liver, PPARα is involved in microsomal, peroxisomal and mitochondrial fatty acid oxidation, fatty acid binding and activation, lipoprotein metabolism (e.g., lipoprotein lipase), gluconeogenesis and triglyceride and LD synthesis and degradation, among others [60]. Outside the liver, PPARα, which is expressed in a variety of tissues as described above, has similar roles. Gemfibrozil-induced activation of PPARα in the respective tissue also results in increased fatty acid uptake [61], which may lead to increased intracellular LD accumulation. Based on this, it can be assumed that gemfibrozil as a ligand for PPAR-α in pre-damaged tissue can lead to an aggravation of the underlying disease, for example diabetic nephropathy or pulmonary pre-disease.

Gemfibrozil, when prescribed, is taken orally in tablet form. It is absorbed into the blood via the gastrointestinal tract, where it is bound to albumin in 97% and thus reaches its target organs. As described earlier, it can cross the blood–brain barrier [6] and thus acts in all tissues of the body where PPARα is expressed as a target receptor. Thus, it can be assumed that gemfibrozil can induce additional effects and changes beyond its intended action as a lipid-lowering agent in the blood. In this regard, the results presented here should be put in context and viewed with caution as they are in vitro results in cell culture. Nevertheless, they seem to show a new property of the drug, which needs to be underlined and proven in further studies. Especially due to the fact that patients treated with gemfibrozil usually have additional comorbidities, the results of this study show that this drug should be prescribed and used with caution. In view of the cell lines investigated, conclusions can be drawn in this relation to patients with known diabetes mellitus, which may be at increased risk for renal dysfunction with gemfibrozil comedication. Additionally, patients with pre-existing pulmonary conditions, such as tuberculosis, cystic fibrosis or even lung carcinoma, may be more likely to suffer harm than benefit from gemfibrozil treatment. In contrast, gemfibrozil in comedication may have a protective effect on neurodegenerative diseases, such as AD.

## 4. Materials and Methods

### 4.1. Chemicals, Reagents, Standards

Further information and a detailed list of used chemicals, reagents and standards in this study can be found in the Supplementary Materials.

### 4.2. Drug Preparation

Gevilon © (Pfizer, Berlin, Germany) as a tablet was diluted in ethanol and vortexed after crushing. This was followed by 80 min of sonication., to create a stock solution. The stock solution was stored at 4 °C and prior to each incubation, the solution was re-sonicated for 15 min. The final concentration of gemfibrozil solution used was 100 µmol/L. This concentration of gemfibrozil was selected on the calculation of the peak plasma concentration of 20 mg/L of gemfibrozil in the human body (corresponding to 79.89 µmol/L) and was selected in line with multiple other cell incubation studies using gemfibrozil [15,16].

### 4.3. Cell culture and Gemfibrozil Treatment

Human neuroblastoma SH-SY5Y wild-type (wt) cells and human embryonic kidney wt (HEK) cells were cultivated in Dulbecco’s Modified Eagle Medium (DMEM, Thermo Fisher, Waltham, MA, USA), containing 10% fetal calf serum (FCS, GE Healthcare Life Sciences, Chalfont St. Giles, UK) and for SH-SY5Y additionally 0.1% non-essential amino acids (MEM), at 37 °C in humified incubator and 5% CO_2_. Calu-3 wt cells, a lung cancer cell line, were cultivated at 37 °C and 5% CO_2_ in DMEM-F12 (Thermo Fisher, Waltham, MA, USA) and 0.1 mM Penicillin/Streptomycin. Cells were cultivated to a confluence of 90%. To exclude the potential effect of gemfibrozil on lipids being also present in FCS, the FCS content in DMEM/DMEM-F12 was reduced to 1% 16 h prior incubation. The different cell lines were 72 h long-term incubated with 100 μmol/L gemfibrozil or a solvent control (ethanol) every 24 h.

### 4.4. Sample Preparation

To harvest the cells after 72 h, they were washed twice with ice-cold HPLC-grade water and the conditioned medium was removed for the use of cell viability (see below). Then, they were taken up in 180 µL water to mechanically homogenate them via Minilys (Peglab, Erlangen, Germany) for 60 s on maximum intensity. In the following, Bicinchoninic acid assay according to Smith et al. was used to measure the protein content of the homogenized samples [62]. In order to perform a homogeneous mass spectrometry study, care was taken to ensure that the protein content within gemfibrozil and its control did not exceed more than 10%. Homogenated samples were adjusted to a protein amount of 10 mg/mL in HPLC-grade water.

The LDH cytotoxicity assay from Roche (Basel, Switzerland) was used according to the protocol of the manufacturer to prevent increased cell toxicity from gemfibrozil or the solvent control. Lactate dehydrogenase (LDH) is an enzyme in the cytoplasm which is rapidly released into the extracellular space when the plasma membrane is damaged [63], so a good marker for cell viability. Care was taken to ensure that cell toxicity did not exceed more than 6%. Cell viability of each cell line with gemfibrozil and its solvent control is shown in Supplementary Figure S1.

### 4.5. Lipid Extraction

The solid/liquid lipid extraction method used in this study follows the protocol described in detail in Grimm et al. [64]. As a short summary: a 96-deep well plate (Fisher Scientific) is covered by a 96-well filter plate (0.45 µm; Merck). Circles of Whatman blotting paper with a diameter of 6 mm were placed into the wells of the filter plate. A standard mixture was added on these Whatman papers followed by 10 µL of each prepared sample (described in sample preparation). After drying the samples under a nitrogen flow (1–2 bar) for 45 min, 20 µL of 5% PITC (*v*/*v*) diluted in ethanol / water / pyridine (1:1:1, *v*/*v*/*v*) were added to the wells and incubated for 20 min at room temperature. Samples are dried another time for 45 min under nitrogen. Then, lipids are extracted by using 300 µL 4.93 mM ammonium acetate in methanol and shaking the plate for 30 min at 450 rpm on a plate shaker (IKA, Staufen, Germany). By centrifugation for 2 min at 500 xg the samples were replaced into the 96-deep well plate. In the following, the samples were diluted with 600 µL 5 mM ammonium acetate in methanol/water (97:3, *v*/*v*). The plate gets covered by a silicone mat and shakes for further 2 min at 450 rpm at room temperature. Then, the samples were analyzed by mass spectrometry.

### 4.6. Targeted Shotgun Mass Spectrometry

The mass spectrometric analysis of lipids extracted from a 100 μg sample using the solid–liquid lipid extraction method described above has already been described in detail [32,65]. In this study a 4000-quadrupole linear-ion trap (QTrap) equipped with a Turbo Spray ion source (AB SCIEX, Darmstadt, Germany) was used to measure different species of diacyl-phosphatidylcholines (PC aa), phosphatidylcholine-plasmalogens (PC ae), lyso-phosphatidylcholines (Lyso-PC), carnitines (C), sphingomyelins (SM) and triacylglycerides (TAG). Using the Analyst 1.4.2 software (AB SCIEX, Darmstadt, Germany) with the help of an autosampler of the Agilent HPLC 1200 (Santa Clara, CA, USA) the detection of different lipid species was conducted in triplicates. Lipid analysis was performed in positive mode using the parameters as described in [32,65]. The used mass spectrometry-method in this study was validated previously in Lauer et al. from 2021 [32]. For further information about the used setting with the main validation parameters please see in the Supplementary Table S5.

### 4.7. Statistical Analysis

The Analyst 1.4.2. Software from AB Sciex was used to extract counts per second for each MRM pair. Each lipid was then normalized to its respective internal lipid class standard and then the mean values per duplicate were determined for each lipid/standard ratio per sample (the raw data obtained from mass spectrometry are listed in the Supplementary Tables S1–S4). This was followed by accounting for the relative changes compared to the solvent control, which were then expressed as percentages in the corresponding bar graphs. The students *t*-test was used as a statistical test to calculate the significance levels between the gemfibrozil-treated cells and the solvent-treated control for each cell line (see volcano plots and bar charts). For further detailed group analysis we used a two-factor ANOVA followed by Tukey post hoc test to obtain the interaction effects of each cell line with each solvent control incubated. Prior to this, we conducted the Levene-Test from the “car”-Package from R (John Fox and Sanford Weisberg (2019). An {R} Companion to Applied Regression, Third Edition. Thousand Oaks CA: Sage. URL: https://socialsciences.mcmaster.ca/jfox/Books/Companion/ (accessed on 15 December 2022)) to obtain the homogeneity of variance in our ANOVA model. These results are shown in the Supplementary Materials. We chose volcano plots to represent each lipid within its lipid class. For this purpose, “R” (R Core Team 2020; Vienna, Austria; https://www.R-project.org/ (accessed on 23 November 2022)) was used for statistical analysis and the two-tailed Student’s *t*-test was used to calculate the *p*-value. The calculated relative changes compared to the solvent control on the abscissa (represented as the logarithm of the percentage fold change) were plotted logarithmically against the corresponding *p*-value on the ordinate. The mean standard error of the mean (SEM) calculated for each lipid species, respectively, is represented by the two vertical lines in the volcano diagram. The volcano plots were created with the R package “enhancedVolcano” (Kevin Blighe, Sharmila Rana and Myles Lewis (2020). Version 1.6.0. https://github.com/kevinblighe/EnhancedVolcano; accessed on 17 September 2022). The Supplementary Table S1 lists all raw data obtained for SH-SY5Y, HEK, and Calu-3 cells treated with gemfibrozil.

## Figures and Tables

**Table 1:** Overview of all lipid classes examined. The percentage values in total for SH-SY5Y, HEK and Calu-3 cells treated with gemfibrozil compared to the solvent-treated control group are shown. In each case, the relative x-fold change with their standard error of the mean (SEM) in lipid species is shown. If statistical significance was reached, this is written underneath. A detailed list of all measured lipid data with additionally group analysis using the ANOVA test following by the Tukey post hoc test and a determination of homogenous groups for each lipid class can be found in the Supplementary Materials (see Supplementary Figure S2A–C and Supplementary Tables S6–S11).

Phospholipid Class (in Total)	SH-SY5Y	HEK	Calu-3
Phosphatidylcholine	103.0% ± 12.7	118.8% ± 5.4	127.6% ± 7.5
			*p* = 0.0127
Phosphatidylcholine plasmalogens	129.9% ± 10.0	98.1% ± 4.1	84.4% ± 6.3
Lyso-phosphatidylcholine	121.0% ± 11.9	126.8% ± 6.1	143.2% ± 9.9
		*p* = 0.0062	*p* = 0.0172
Sphingomyelin	126.6% ± 12.2	121.0% ± 3.7	110.8% ± 6.9
		*p* = 0.047	
Acyl-carnitine	130.7% ± 4.9	100.5% ±5.9	102.2 ± 7.4
Triacylglyceride	170.3% ± 11.1	272.1% ± 14.3	448.1% ± 10.8
	*p* ≤ 0.001	*p* ≤ 0.001	*p* ≤ 0.001

## Data Availability

Not applicable.

## Supplementary Materials

The following supporting information can be downloaded at: https://www.mdpi.com/article/10.3390/ijms24032972/s1.
