# Supplementary material for: Gemfibrozil-Induced Intracellular Triglyceride Increase in SH-SY5Y, HEK and Calu-3 Cells
Source: Int J Mol Sci. 2023 Feb 3;24(3):2972. doi: 10.3390/ijms24032972 (PMC9917468; doi:10.3390/ijms24032972)
Supplement: Supplementary file 1 [file ijms-24-02972-s001.zip › ijms-2158195_supplementary materials.pdf]

Supplementary Material

Gemfibrozil-induced intracellular Triglyceride increase in SH-SY5Y, HEK and Calu-3 cells

Cornel Manuel Bachmann [1,†], Daniel Janitschke [1,†] , Anna Andrea Lauer [1,2] , Tobias Erhardt [3], Tobias Hartmann [1,4] , Marcus Otto Walter Grimm [1,2,4,*,‡] and Heike Sabine Grimm [1,2,*,‡]

[1] Experimental Neurology, Saarland University, 66421 Homburg, Germany

[2] Nutrition Therapy and Counseling, Campus Rheinland, SRH University of Applied Health Sciences, 51377 Leverkusen, Germany

[3] Physical Therapy, Campus Karlsruhe, SRH University of Applied Health Sciences, 76185 Karlsruhe, Germany

[4] Deutsches Institut für DemenzPrävention (DIDP), Saarland University, 66421 Homburg, Germany

* Correspondence: marcus.grimm@uks.eu (M.O.W.G.); heike.grimm.extern@srh.de (H.S.G.)

† These first authors contributed equally to this work.

‡ These senior authors contributed equally to this work.

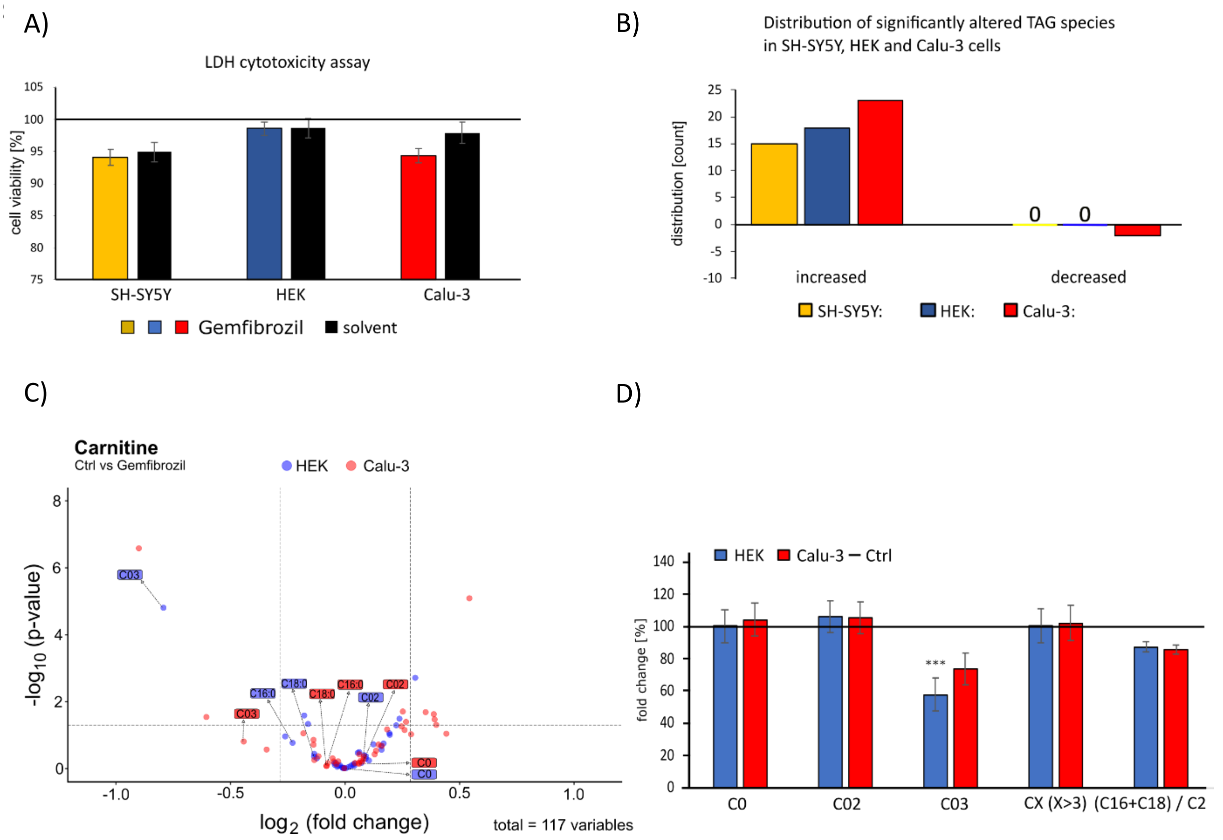

Supplemental figure S1: A) Investigation of cell viability after incubation of SH-SY5Y, HEK and Calu-3 cells with 100 µmol/l gemfibrozil for 72 h (0 + 24 + 48 h) via cytotoxicity detection assay. Care was taken not to exceed 6% of cytotoxicity. **B)** A bar chart shows the distribution of significantly altered TAG-species after gemfibrozil treatment in SH-SY5Y, HEK and Calu-3 cells. All species were considered that fell below the significance level of $p = 0.05$. On the left side all significantly increased species for each cell type are shown. On the right side are all significantly decreased species illustrated. **C)** Changed carnitine levels in HEK and Calu-3 cells after gemfibrozil treatment compared to cells treated with the solvent control (ethanol) are illustrated in the volcano plot. The fold change (x-axis) of each of the 41 examined carnitine species (dots) was plotted against the corresponding logarithmic p-value (y-axis). A detailed structure of the volcano plot is explained in the legend of figure 1. **D)** The bar chart shows the changes in C0, C2, C3, CX (X > 3) and (C16 + C18)/C2 ratio in gemfibrozil treated HEK and Calu-3 cells compared to cells treated with the solvent control (ethanol), which is represented as the horizontal line and set as 100%. The obtained data were calculated by using the students t-test comparing gemfibrozil-treated cells against the control of each cell line.

A)

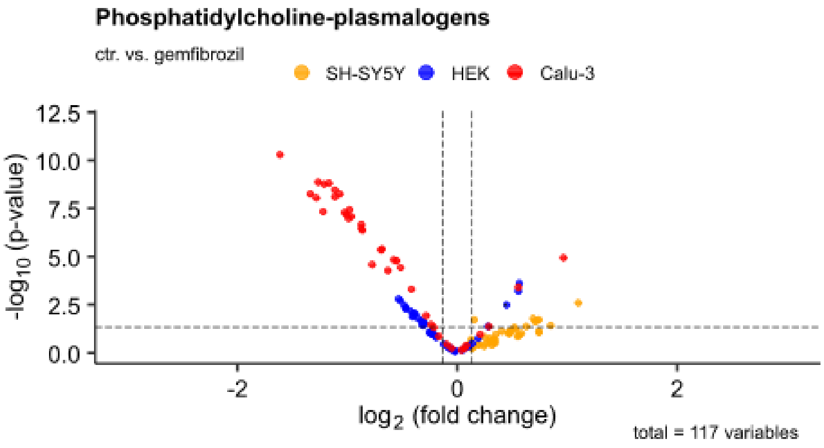

B)

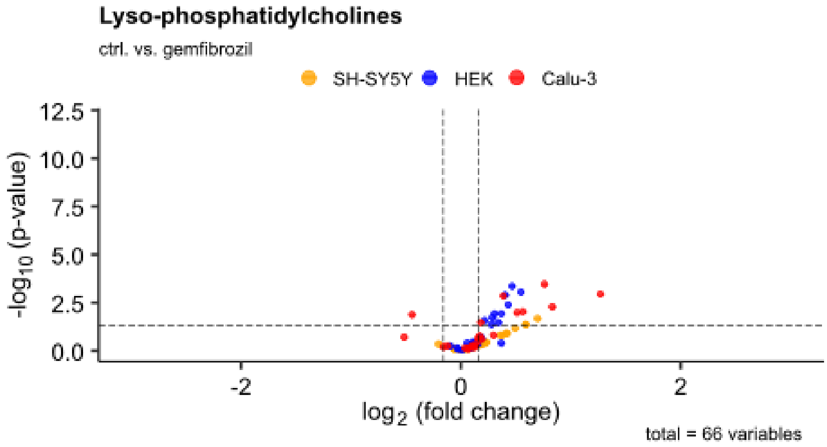

C)

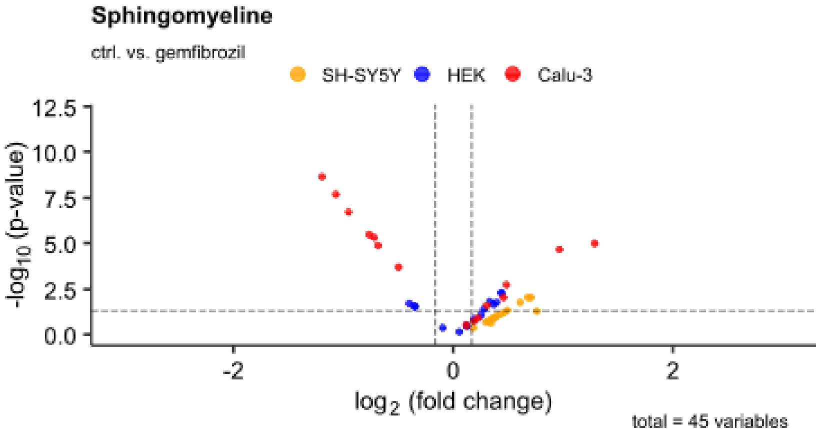

Supplemental figure S2: A) - C) changed Phosphatidylcholine-plasmalogen (PCae), Lyso-phosphatidylcholine (Lyso-PC) and Sphingomyelin (SM) levels in SH-SY5Y, HEK and Calu-3 cells after gemfibrozil treatment compared to cells treated with the solvent control (ethanol) are illustrated in the volcano plots (A – C), where the fold change (x-axis) of each of the examined lipid species (dots) was plotted against the corresponding logarithmic p-value (y-axis). All three cell lines are shown in one vol-cano plot with three different colors: SH-SY5Y in orange, HEK in blue and Calu-3 in red.

4.1. Chemicals, Reagents, Standards

HPLC-grade pyridine, phenyl isothiocyanate (PITC) and ammonium acetate used for lipid extraction were purchased from Merck (Darmstadt, Germany). All other chemicals including high performance liquid chromatography (HPLC)-grade water, ethanol, and methanol were purchased from Fisher Scientific (Schwerte, Germany), if not stated otherwise. Gemfibrozil was friendly provided in form of Gevilon© 600 mg (from Pfizer) by the Kronen-Apotheke, 09212 Limbach-Oberfrohna, Germany. For normalization the following standards from Avanti Polar Lipids were used: 06:0 PC (DHPC), 19:0 Lyso PC, 06:0 SM (d18:1/6:0), and Splash II Lipidomix Mass Spec Internal Standard. The carnitine standards octanoyl-L-carnitine d3 and palmitoyl-L-carnitine d3 were purchased from Supelco Analytical.

4.6. Targeted Shotgun Mass Spectrometry

Table S5 lists all validation parameters required for shotgun mass spectrometry. A detailed description of the validation and their respective origin data can be found in Lauer et al. [32].

Table S5: Overview of the used validation parameters for Mass spectrometry analysis in this study. For the data obtained in this work, the experimental conditions of Lauer et. al. [32] were adopted. The focus was on average extraction efficiency, linearity for each lipid class, intra- and inter-day variance, and potential matrix effects.

**Validation parameter**	**result**
Average extraction efficiency	> 80.7 %
Intra-day variance (extraction)	3.9 %
Linearity of PC aa	$R^2 = 0.98$
Linearity of PC ae	$R^2 = 0.97$
Linearity of Lyso-PC	$R^2 = 0.96$
Linearity of SM	$R^2 = 0.98$
Linearity of C16	$R^2 = 0.99$
Linearity of TAG	$R^2 = 0.99$
Intra- and inter-day variance of lipid detection	6.5 %
Potential matrix effects	< 3.2 %

Group analysis

Table S6: Results of group analysis of the raw data of **triglycerides** (TAG) total. In this analysis we calculated via a Two-Factor ANOVA (Factor 1 treatment (control vs gemfibrozil) and Factor 2 cell line (Calu-3, HEK and SH-SY5Y). Following this we calculated the p-values of each combination via the post hoc test TukeyHSD (calculated p-values shown in the table below). Prior to this we used the LeveneTest to determine if the homogeneity of variances was given for this model (see p-value LeveneTest: p = 0.5558).

	SH-SY5Y ctrl	SH-SY5Y gemfi	HEK ctrl	HEK gemfi	Calu-3 ctrl	Calu-3 gemfi
SH-SY5Y ctrl	-	**$p = 6.06x10^{-4}$**	$p = 8.57x10^{-1}$	**$p = 9.72x10^{-11}$**	**$p = 8.09x10^{-11}$**	**$p = 8.16x10^{-13}$**
SH-SY5Y gemfi	**$p = 6.06x10^{-4}$**	-	$p = 1.23x10^{-2}$	**$p = 1.10x10^{-4}$**	**$p = 9.00x10^{-5}$**	**$p = 8.16x10^{-13}$**
HEK ctrl	$p = 8.57x10^{-1}$	$p = 1.23x10^{-2}$	-	**$p = 1.31x10^{-9}$**	**$p = 1.08x10^{-9}$**	**$p = 8.15x10^{-13}$**
HEK gemfi	**$p = 9.72x10^{-11}$**	**$p = 1.10x10^{-4}$**	**$p = 1.31x10^{-9}$**	-	$p = 9.99x10^{-1}$	**$p = 8.33x10^{-13}$**
Calu-3 ctrl	**$p = 8.09x10^{-11}$**	**$p = 9.00x10^{-5}$**	**$p = 1.08x10^{-9}$**	$p = 9.99x10^{-1}$	-	**$p = 8.34x10^{-13}$**
Calu-3 gemfi	**$p = 8.16x10^{-13}$**	**$p = 8.16x10^{-13}$**	**$p = 8.15x10^{-13}$**	**$p = 8.33x10^{-13}$**	**$p = 8.34x10^{-13}$**	-

Table S7: Results of group analysis of the raw data of **acylcarnitines** (C) total. In this analysis we calculated via a Two-Factor ANOVA (Factor 1 treatment (control vs gemfibrozil) and Factor 2 cell line (Calu-3, HEK and SH-SY5Y). Following this we calculated the p-values of each combination via the post hoc test TukeyHSD (calculated p-values shown in the table below). Prior to this we used the LeveneTest to determine if the homogeneity of variances was given for this model (see p-value LeveneTest: p = 0.2301).

	SH-SY5Y ctrl	SH-SY5Y gemfi	HEK ctrl	HEK gemfi	Calu-3 ctrl	Calu-3 gemfi
SH-SY5Y ctrl	-	$p = 9.99x10^{-1}$	$p = 8.90x10^{-1}$	$p = 9.10x10^{-1}$	**$p = 6.54x10^{-3}$**	**$p = 2.30x10^{-3}$**
SH-SY5Y gemfi	$p = 9.99x10^{-1}$	-	$p = 9.21x10^{-1}$	$p = 9.39x10^{-1}$	**$p = 3.08x10^{-3}$**	**$p = 1.01x10^{-3}$**
HEK ctrl	$p = 8.90x10^{-1}$	$p = 9.21x10^{-1}$	-	$p = 9.99x10^{-1}$	**$p = 1.53x10^{-4}$**	**$p = 4.66x10^{-5}$**
HEK gemfi	$p = 9.10x10^{-1}$	$p = 9.39x10^{-1}$	$p = 9.99x10^{-1}$	-	**$p = 1.85x10^{-4}$**	**$p = 5.64x10^{-5}$**
Calu-3 ctrl	**$p = 6.54x10^{-3}$**	**$p = 3.08x10^{-3}$**	**$p = 1.53x10^{-4}$**	**$p = 1.85x10^{-4}$**	-	$p = 9.99x10^{-1}$
Calu-3 gemfi	**$p = 2.30x10^{-3}$**	**$p = 1.01x10^{-3}$**	**$p = 4.66x10^{-5}$**	**$p = 5.64x10^{-5}$**	$p = 9.99x10^{-1}$	-

Table S8: Results of group analysis of the raw data of **phosphatidylcholines** (PC aa) total. In this analysis we calculated via a Two-Factor ANOVA (Factor 1 treatment (control vs gemfibrozil) and Factor 2 cell line (Calu-3, HEK and SH-SY5Y). Following this we calculated the p-values of each combination via the post hoc test TukeyHSD (calculated p-values shown in the table below). Prior to this we used the LeveneTest to determine if the homogeneity of variances was given for this model (see p-value LeveneTest: p = 0.2834).

	SH-SY5Y ctrl	SH-SY5Y gemfi	HEK ctrl	HEK gemfi	Calu-3 ctrl	Calu-3 gemfi
SH-SY5Y ctrl	-	$p = 5.35x10^{-1}$	$p = 9.99x10^{-1}$	$p = 5.63x10^{-1}$	$p = 2.86x10^{-1}$	**$p = 4.77x10^{-3}$**
SH-SY5Y gemfi	$p = 5.35x10^{-1}$	-	$p = 5.86x10^{-1}$	p = 1.000	$p = 9.97x10^{-1}$	$p = 2.42x10^{-1}$
HEK ctrl	$p = 9.99x10^{-1}$	$p = 5.86x10^{-1}$	-	$p = 6.15x10^{-1}$	$p = 3.18x10^{-1}$	**$p = 4.83x10^{-3}$**
HEK gemfi	$p = 5.63x10^{-1}$	p = 1.000	$p = 6.15x10^{-1}$	-	$p = 9.96x10^{-1}$	$p = 2.23x10^{-1}$
Calu-3 ctrl	$p = 2.86x10^{-1}$	$p = 9.97x10^{-1}$	$p = 3.18x10^{-1}$	$p = 9.96x10^{-1}$	-	$p = 4.84x10^{-1}$
Calu-3 gemfi	**$p = 4.77x10^{-3}$**	$p = 2.42x10^{-1}$	**$p = 4.83x10^{-3}$**	$p = 2.23x10^{-1}$	$p = 4.84x10^{-1}$	-

Table S9: Results of group analysis of the raw data of **phosphatidylcholines-plasmalogens** (PC ae) total. In this analysis we calculated via a Two-Factor ANOVA (Factor 1 treatment (control vs gemfibrozil) and Factor 2 cell line (Calu-3, HEK and SH-SY5Y). Following this we calculated the p-values of each combination via the post hoc test TukeyHSD (calculated p-values shown in the table below). Prior to this we used the LeveneTest to determine if the homogeneity of variances was given for this model. (See p-value LeveneTest: p = 0.3274).

	SH-SY5Y ctrl	SH-SY5Y gemfi	HEK ctrl	HEK gemfi	Calu-3 ctrl	Calu-3 gemfi
SH-SY5Y ctrl	-	$p = 3.47x10^{-1}$	**$p = 3.18x10^{-8}$**	**$p = 2.61x10^{-8}$**	**$p \leq 1.88x10^{-12}$**	**$p = 7.99x10^{-11}$**
SH-SY5Y gemfi	$p = 3.47x10^{-1}$	-	**$p = 1.18x10^{-5}$**	**$p = 9.58x10^{-6}$**	**$p = 1.47x10^{-10}$**	**$p =1.81x10^{-8}$**
HEK ctrl	**$p = 3.18x10^{-8}$**	**$p = 1.18x10^{-5}$**	-	$p = 9.99x10^{-1}$	**$p = 1.16x10^{-2}$**	$p = 3.58x10^{-1}$
HEK gemfi	**$p = 2.61x10^{-8}$**	**$p = 9.58x10^{-6}$**	$p = 9.99x10^{-1}$	-	**$p = 1.38x10^{-2}$**	$p = 3.94x10^{-1}$
Calu-3 ctrl	**$p \leq 1.88x10^{-12}$**	**$p = 1.47x10^{-10}$**	**$p = 1.16x10^{-2}$**	**$p = 1.38x10^{-2}$**	-	$p = 6.33x10^{-1}$
Calu-3 gemfi	**$p = 7.99x10^{-11}$**	**$p =1.81x10^{-8}$**	$p = 3.58x10^{-1}$	$p = 3.94x10^{-1}$	$p = 6.33x10^{-1}$	-

Table S10: Results of group analysis of the raw data of **Lyso-phosphatidylcholines** (Lyso-PC) total. In this analysis we calculated via a Two-Factor ANOVA (Factor 1 treatment (control vs gemfibrozil) and Factor 2 cell line (Calu-3, HEK and SH-SY5Y). Following this we calculated the p-values of each combination via the post hoc test TukeyHSD (calculated p-values shown in the table below). Prior to this we used the LeveneTest to determine if the homogeneity of variances was given for this model (see p-value LeveneTest: p = 0.1611).

	SH-SY5Y ctrl	SH-SY5Y gemfi	HEK ctrl	HEK gemfi	Calu-3 ctrl	Calu-3 gemfi
SH-SY5Y ctrl	-	$p = 6.89x10^{-1}$	**$p = 1.11x10^{-5}$**	**$p = 8.29x10^{-10}$**	$p = 8.99x10^{-1}$	**$p = 6.38x10^{-3}$**
SH-SY5Y gemfi	$p = 6.89x10^{-1}$	-	**$p = 7.15x10^{-4}$**	**$p = 3.65x10^{-8}$**	$p = 9.99x10^{-1}$	$p = 1.84x10^{-1}$
HEK ctrl	**$p = 1.11x10^{-5}$**	**$p = 7.15x10^{-4}$**	-	**$p = 4.06x10^{-2}$**	**$p = 1.88x10^{-4}$**	$p = 2.97x10^{-1}$
HEK gemfi	**$p = 8.29x10^{-10}$**	**$p = 3.65x10^{-8}$**	**$p = 4.06x10^{-2}$**	-	**$p = 9.33x10^{-9}$**	**$p = 8.32x10^{-5}$**
Calu-3 ctrl	$p = 8.99x10^{-1}$	$p = 9.99x10^{-1}$	**$p = 1.88x10^{-4}$**	**$p = 9.33x10^{-9}$**	-	$p = 7.44x10^{-2}$
Calu-3 gemfi	**$p = 6.38x10^{-3}$**	$p = 1.84x10^{-1}$	$p = 2.97x10^{-1}$	**$p = 8.32x10^{-5}$**	$p = 7.44x10^{-2}$	-

Table S11: Results of group analysis of the raw data of **Sphingomyelines** (SM) total. In this analysis we calculated via a Two-Factor ANOVA (Factor 1 treatment (control vs gemfibrozil) and Factor 2 cell line (Calu-3, HEK and SH-SY5Y). Following this we calculated the p-values of each combination via the post hoc test TukeyHSD (calculated p-values shown in the table below). Prior to this we used the LeveneTest to determine if the homogeneity of variances was given for this model. (See p-value LeveneTest: p = 0.1624).

	SH-SY5Y ctrl	SH-SY5Y gemfi	HEK ctrl	HEK gemfi	Calu-3 ctrl	Calu-3 gemfi
SH-SY5Y ctrl	-	$p = 1.47x10^{-1}$	$p = 6.61x10^{-2}$	**$p =5.26x10^{-4}$**	**$p = 1.10x10^{-3}$**	**$p = 1.24x10^{-4}$**
SH-SY5Y gemfi	$p = 1.47x10^{-1}$	-	$p = 9.98x10^{-1}$	$p = 2.68x10^{-1}$	$p = 3.96x10^{-1}$	$p = 1.06x10^{-1}$
HEK ctrl	$p = 6.61x10^{-2}$	$p = 9.98x10^{-1}$	-	$p = 4.99x10^{-1}$	$p = 6.56x10^{-1}$	$p = 2.42x10^{-1}$
HEK gemfi	**$p =5.26x10^{-4}$**	$p = 2.68x10^{-1}$	$p = 4.99x10^{-1}$	-	$p = 9.99x10^{-1}$	$p = 9.97x10^{-1}$
Calu-3 ctrl	**$p =1.10x10^{-3}$**	$p = 3.96x10^{-1}$	$p = 6.56x10^{-1}$	$p = 9.99x10^{-1}$	-	$p = 9.78x10^{-1}$
Calu-3 gemfi	**$p = 1.24x10^{-4}$**	$p = 1.06x10^{-1}$	$p = 2.42x10^{-1}$	$p = 9.97x10^{-1}$	$p = 9.78x10^{-1}$	-
